# Numerous Paragraphs

**Published:** 1894-12

**Authors:** 


### Medical News and Miscellany.

**Dr. W. B. Anderson** has removed from Taylor to Brownwood.

**Dr. Wm. Wilke**, of San Antonio, suicided by cutting his throat. Cause, ill health. Aged 60.

**Dr. Samuel E. Milliken** has been appointed Surgeon to the Randall's Island Hospital, New York.

**Dr. Tiffany**, of Baltimore, succeeds Dr. Miles as Surgeon in the Medical Department of Tulane University, says the *New York Medical Record.*

**Wanted**—A co-partnership with a physician doing a paying and growing practice in a growing town. Address Dr. W., care TEXAS MEDICAL JOURNAL, Austin, Texas.

**For Sale**—My residence, centrally located in a town of 3000; well established practice worth $3500 cash annually. Residence and practice goes for $2500—$1000 cash, the balance on easy terms. For particulars address Dr. Boze, care TEXAS MEDICAL JOURNAL.

---

**Holiday Excursions to the South-East.**—On December 20th, 21st and 22d, 1894, the International Route will, as usual, have on sale Holiday Excursion tickets to South-Eastern States, including St. Louis, Louisville, Cincinnati, Memphis and New Orleans, at rate of one fare for round trip, tickets limited to 30 days for return. Call on nearest ticket agent for full information.

D. J. PRICE, A. G. P. A.

---

**Died.**—At Huntsville, Texas, November 17th, ult., Miss Kittie Walker, daughter of Dr. W. W. Walker, of Shulenburg, and sister of Drs. E. R. and W. H. Walker. She was attending school at Huntsville, and died of typhlitis. The JOURNAL is pained to chronicle the too early fading of this pretty young flower. She was just seventeen, and was the pride and joy of her father's household. Our tenderest sympathies go out to the bereaved family.

---

**For Sale**—Residence and practice in a flourishing small town in the German and Bohemian settlement, where practice is mostly cash. Business about $2000 a year. House of 5 rooms, situated on a block of ground; good out-houses, splendid barn and good fruit orchard. Price, $1000, half cash. The money can be made out of the practice the first year, by a good doctor. Reason for selling, to remove to a city. For particulars address E. R. W., care TEXAS MEDICAL JOURNAL, Austin, Texas.

---

**Medical Consultation Book.**—This great work, by Dr. Hachenburg, which has received very high commendations at the hands of reviewers, and which was sold at $7.50 per volume, can now be had in club with the JOURNAL for $2.50,—the two for $4.50,—by addressing this office. By a special arrangement with the publisher, we are enabled to offer the few remaining unsold copies of this splendid work at just 33⅓ cents on the dollar, to close them out. This is the original revised and unabridged work. Here is a rare chance, doctor.

---

**Economy** and efficiency in management have characterized the administration of the Texas Health Department. The appropriation for all purposes for the past four years has been only $45,000 a year, and in no instance has the full amount of appropriation been expended. It will be seen from the State Health Officers report, extracts from which are published in this issue, that that officer estimates the expenses for all purposes for the next two years, at only $42,000 a year. This is less money, we venture, then is expended by any Board of Health in any State.

---

**Death of Mrs. Garwood.**—The JOURNAL is pained to announce the death of Mrs. Garwood, wife of Dr. Alonzo Garwood, of New Braunfels. This most estimable lady died very recently, at her home. We had not heard of her illness, and hence the announcement comes with a greater shock. She was a daughter of the late Senator Geo. Pfeuffer, and was a most accomplished lady. She was a tender and careful mother, a most examplary wife, and the charm and center of a large circle of devoted, loving friends. Theirs was a lovely, happy home, and her death is most distressing. She leaves two little children, George and Lucile, the pride and delight of the home now so sadly bereft. Our tenderest sympathy goes out to our friend, the distressed husband, and to the sorrowing familes.

---

**Dr. E. P. Becton.**—The many friends of this gifted and distinguished Texas physician will be pleased to learn that he is to succeed Dr. Frank Rainey as superintendent of the State Institute for the Blind, at Austin, and will take charge upon the advent of the new administration. Dr. Becton is one of the most popular physicians in Texas, and acknowledged to be one of the best. He is a natural orator, and at our State Medical Association meetings is one of the standbys and main reliances for a speech. Dr. Rainey, who has been in continuous charge of the Institute for the Blind for twenty years, and has made a most excellent record, upon his retirement will engage in the book and stationery business in Austin. Many of his friends will regret his retirement, and we are sure there will be tears amongst the pupils, to whom he has so long been like a father, and the old employes, who were much attached to him.

---

**Asylum Data.**—The reports of Superintendents White, of the Austin branch, and Preston, of the Terrell branch, State Lunatic

Asylum, make the following showing. That of Superintendent Barker, of the San Antonio asylum, not to hand:

| | Terrell. | Austin. |
|---|---|---|
| Remaining in asylum, Oct. 31, 1893 | 783 | 659 |
| Admitted during the year | 239 | 126 |
| Whole number under treatment | 1022 | 782 |
| Discharged restored | 106 | 74 |
| Discharged improved | 49 | 4 |
| Discharged unimproved | 2 | 4 |
| Died | 52 | 25 |
| Remaining, Oct. 31, 1894 | 813 | 675 |
| Percentage of discharges to No. treated | 15.3 | 4.04 |
| Percentage of deaths to No. treated | 5.08 | 3.19* |
| Cost of maintenance per capita | —— | $150.48† |

The above is a most creditable showing. For comparison we give herewith the death rate at the Austin asylum since 1886. In that year it was 10.62, then for consecutive years, 5.61, 4.95, 3.22, 4.95, 4.50, 4.40, 3.69. We regret we have not Dr. Preston's full report at hand to make a similar comparison. Nor any report from Dr. Barker.

The per capita cost during the last four years, at Austin asylum, has decreased from $180.34 to $150.48.

It may be interesting to readers to know that at the Austin asylum, during the year, there were produced farm products to

| | |
|---|---|
| the value of | $5,627 96 |
| Garden products | 2,794 90 |
| Dairy products | 3,658 75 |
| A total of | $12,081 61 |

---

**The Austin District Medical Society** will meet in the K. of P. Hall, Austin, Texas, at 10 a. m., Thursday, December 20, 1894. A cordial invitation is extended to all members of the regular medical profession. Good papers, able discussions, election of officers, followed by a banquet at night. Come and be one of us. S. E. HUDSON, Secretary.

---

**The Free Bath.**—The Superintendent of the Hot Springs Reservation in his report says:

"It is with much personal gratification that I am now able to say that the free bath house is kept in as thoroughly respectable

---

* Lowest death rate ever reported.
† Lowest per capita ever reported.

and cleanly condition as any bath house or hospital in the country.

"If it is charity to cure the helpless indigent people who come to Hot Springs, and who must inevitably die of the diseases with which they are afflicted except for the relief afforded by the use of these baths, and charity is remembered and appreciated by those to whom it is given, then the free bath house at Hot Springs should have a warm place in the hearts of many people to whom it has given back health.

"In most cases the people using these baths are absolutely without the means of procuring medical advice or medicines, and depend entirely on the baths for whatever benefit the waters will give. When with this is understood that the worst cases that come to Hot Springs are indigent persons who find their way to the free bath house, it is wonderful and incredible the number of these that are actually cured. It is true that in many cases a long course of bathing is required, but it is no exaggeration to say that 75 per cent. of all the persons using these baths are absolutely cured, and most of the balance are greatly benefited.

"It should be understood and kept before the public that the capacity of this free bath house is not sufficient to bathe all the indigent persons in the United States. It has two pools for men and two for women, the pools for men being 12 by 12½ ft. each, while the pools for women are only 7 by 12 ft, each. And when it is understood that in this limited space an average of 556 persons have been bathed daily, and that from June 30, 1893, to June 30, 1894, about 203,000 baths have been given, it may be understood also that the house has been run to its capacity.

"I had hoped at this time to give a full half year's report of the operations of the free bath house under the system of issuing tickets only upon written applications, but during part of January the bath house was undergoing repairs and no accurate report could be made for that month.

"The five months, from February to June, are reported here for the purpose of showing the operations of the house as they actually occur.

Table showing number of bathers for five months, February to June, inclusive:

| | Feb'y. | March. | April. | May. | June. |
|---|---|---|---|---|---|
| Daily average of white males | 258 | 321 | 336 | 325 | 202 |
| Daily average of white females | 26 | 56 | 76 | 64 | 43 |
| Daily average of colored males | 108 | 175 | 205 | 127 | 112 |
| Daily average of colored females | 42 | 97 | 133 | 103 | 80 |
| Average number of persons bathed daily | 434 | 649 | 750 | 509 | 437 |

"The matter of discrimination necessary to be exercised in issuing tickets to the free bath house is a matter in which it seems impossible to give entire satisfaction. It may be seen by this report that all possible liberality is observed in making admissions, but those whom we are compelled to refuse, who upon examination do not appear indigent persons, and those who desire to use the free bath house as a matter of economy or convenience, are usually very pronounced in their complaints, and often abusive.

"The wisdom and justness of the rules and regulations under which the house is now operated are amply attested by the number of indigent persons it has cured and restored the means of becoming useful citizens, instead of paupers, as they were before, and the fact that the house is thoroughly clean and respectable and has taken rank as the government free bath house, in place of continuing to be known as the "Mud Hole" as heretofore, is ample consolaton to the Superintendent for all the criticisms that may have been made upon him on account of enforcing the rules under which the house is now operated.

"The house is open to visitors and strangers every Wednesday afternoon from 2 to 6 o'clock, during which time bathing is suspended, and visitors given such information as they may desire."

---

**Asylum Reform.**—Supt. F. S. White, of the Austin branch of the State Lunatic Asylum, in his report says:

"Our asylum methods are in many instances wrong and misleading; their tendency is often to make lunatics, rather than to cure them. In the commodious, magnificent, well kept and beautiful parks of all modern asylums will be seen hundreds of lunatics sitting, day in and day out, in idleness and misery, watched by attendants who take little interest in them further than to be very careful that no one makes his escape.

"The monotony of such a life is something terrible, when we contemplate that by fostering this idleness and monotony the last hope of the recovery of many is being frittered away. I have for years advocated, and still advocate, with all my power, the necessity of some kind of a change that will result beneficially to those most interested, viz., the poor, helpless patients.

"Visit one of the beautiful parks; you will see dozens of men pacing regularly, solemnly, and slowly, the same path backward and forward which they have worn in the ground near where an attendant sits; others are sitting beside the same stately oak whose umbrageous branches have shaded them possibly for years. There they sit for hours, wrapt in the gloom of their own meditations, dead to the world, to themselves, and forming only an atom of the great mass of humanity that has crystallized into a so-called well regulated and modern asylum. These cases are all strong and healthy physically; they eat, drink, sleep, and walk out and in daily. Such is their life; no diversion, no change, nothing save a monotonous routine, without a ray of hope in the distance to brighten the gloomy present.

"If there is a class in the world that needs stimulating, moving, jostling and stirring up, it certainly is the chronic insane. It may be asked, how can this condition be remedied? One word, employment, solves the problem. If land near this asylum were not so valuable, I would recommend the purchase by the State of an additional large tract. Double the amount, at least, now in cultivation here, could be cultivated at a very slight increase in expense to the State. It would only be necessary to add a few more attendants; we already have numbers of patients who would in a short time be only too glad to work on the farm. If land could be bought here at say $50 per acre, it would even then be a good investment for the State.

"With 200 additional acres, we could greatly decrease the annual cost of maintenance, and at the same time give employment to every patient that desired or was able to work; the recovery rate would be increased, more could be cared for during the year, and their life rendered not altogether a blank. Owing, however, to the high price of land, it is not probable that the legislature would think best to add any more to the farm here, but it would be wisdom and economy to purchase a large tract of the best tillable land in the State in some county where crops are comparatively certain, and there establish an asylum farm; erect buildings on the cottage system, with a view of adding more when demanded, colonize the great army of the insane thereon, and make them, to a very large extent, self-sustaining. Thousands of bushels of vegetables can be raised, and the surplus canned for future consumption.

"I believe that for a very small outlay a canning factory can be established here that will pay handsomely, and at the same time add very greatly to the dietary of the patients; corn, okra, tomatoes and beans can be grown in great abundance. If these could be canned successfully, the acreage could be increased and a large supply stored for future use.

"With the constant increase of the insane in Texas, and as a consequence the increased cost of their maintenance so prominent before us, it appears that any plan that would decrease either would be very acceptable. The insane are a part of every State; they are not producers, but consumers; in one sense, they are a necessary burden on every government. They are the debris of a too rapidly developing civilization, but like the barnacles that hang to the ship's hull, they hang on to the body politic, and must be taken care of. The dictates of humanity demand that this unfortunate class be cared for; public policy demands that they be cared for as economically as is compatible with their welfare.

"Texas cannot afford to house her insane in shanties and hovels, neither can she afford to provide them with palaces. A happy medium must be found, which, while being comfortable, safe, etc., is at the same time reasonable as to cost."

